# Stroke-Like Migraine Attacks After Radiation Therapy (SMART) Syndrome Presenting With Recurrent Seizures: A Case Study

**DOI:** 10.7759/cureus.25691

**Published:** 2022-06-06

**Authors:** Neha Panigrahy, Surya Aedma, Matthew Lee

**Affiliations:** 1 Medicine, Carle Illinois College of Medicine, Champaign, USA; 2 Internal Medicine, Carle Foundation Hospital, Urbana, USA; 3 Internal Medicine, Carle Foundation Hospital, Champaign, USA

**Keywords:** diagnostic work up, primary brain tumor, post-radiation malignancy, seizures, smart syndrome

## Abstract

Stroke-like migraine attacks after radiation therapy (SMART) syndrome is a rare, delayed complication of radiation therapy to the brain. We present a case of a 49-year-old female with a past medical history of malignant neoplasm of the brain status following resection and radiation. She initially presented with increased work of breathing. Initial labs and a chest X-ray were suggestive of aspiration pneumonia leading to sepsis. Upon hospitalization, seizure-like activity was noted. Electroencephalogram showed electrographic seizures originating from the left occipital and parietal lobe. She received numerous medications to control the seizures with minimal improvement. Magnetic resonance imaging was performed to characterize the origin of seizures, which showed extensive post-radiation changes including a new meningioma. The patient was subsequently managed with magnesium and Solu Medrol. After this regimen, her condition improved and there were no clinical seizures present.

## Introduction

Stroke-like migraine attacks after radiation therapy (SMART) syndrome is a rare, delayed complication of radiation therapy to the brain. To our knowledge, there has not been a reported association with tumor type, and the presentation may be delayed two to three decades after radiation treatment. SMART syndrome has been described as transient, reversible neurologic dysfunction, most commonly presenting with migraine-like headache, stroke-like focal neurologic deficits, and encephalopathy [1,2]. Characteristic magnetic resonance imaging (MRI) findings include unilateral gyral swelling and gadolinium enhancement, most commonly in the parieto-occipital region [3,4].

Seizures have long been recognized as a common feature of SMART syndrome, with the largest case series finding seizures in 35% (9 of 26) and 64% (23 of 36) patients [5,6]. However, newer reports suggest that seizures and status epilepticus may be more prevalent and argue that seizures may be a cardinal feature of SMART syndrome [7,8]. We describe here a case of SMART syndrome presenting with super-refractory status epilepticus (SRSE) to further elucidate the role of seizures in the rare complication of brain radiotherapy.

## Case presentation

A 49-year-old female with a significant past medical history of seizure disorder (on topiramate 100 mg daily and lacosamide 50 mg), oligodendroglioma status after surgical resection and radiation, hydrocephalus with bilateral contractures, chronic respiratory failure with tracheostomy, non-ventilator dependent, and dysphagia with a gastrostomy (G) tube was admitted in April 2021 for fever and increased work of breathing. Complete blood counts at the time of admission showed a white blood count of 20.21 cmm, hemoglobin level of 9.2 g/dL, hematocrit level of 33.9 L/L, and platelet count of 535 per microliter of blood. Metabolic panel at the time of admission showed sodium at 149 mEq/L, chloride level at 123 mEq/L, creatinine at 1.35 μmol/L, albumin at 2.4 g/dL, and alkaline phosphatase at 143 U/L. EKG showed no acute ischemic changes and chest X-ray showed left lower lobe pneumonia. The patient was managed for sepsis.

On hospital day 10, a family member of the patient noted that she had jerking eye movements, and requested monitoring of seizures. Long-term electroencephalogram (EEG) monitoring showed electrographic seizures from the left occipital and posterior temporal region. She was loaded with Keppra and then started on 1500 mg twice daily with the continuation of Vimpat 200 mg BID and Topamax 200 mg twice a day. The patient was also given 3 mg of Ativan. In the first 13 hours of long-term monitoring, the patient had 26 electrographic seizures. Keppra was changed to 1000 mg every eight hours and Ativan 1 mg every eight hours, with Vimpat 200 mg twice daily and Topamax 200 mg twice daily.

**Figure 1:**
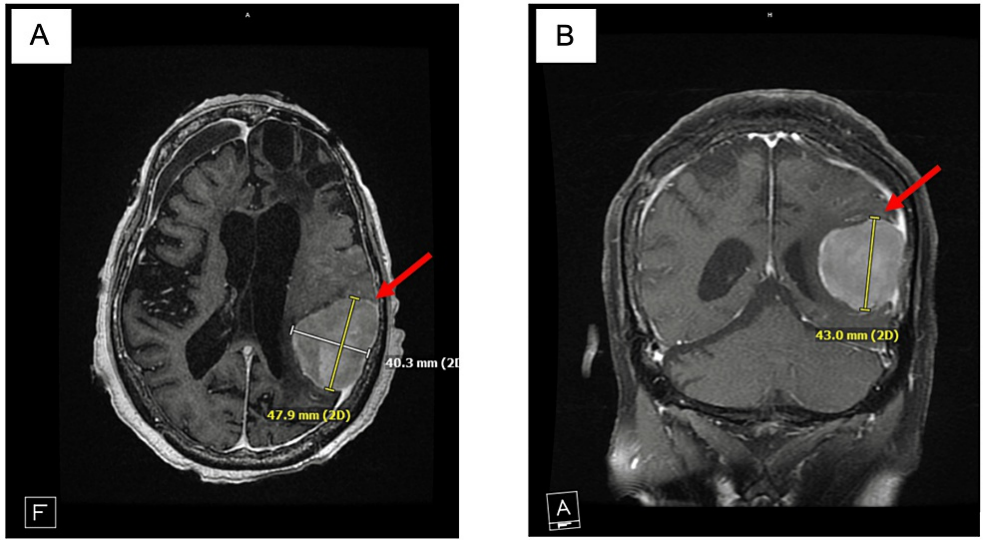
New radiation-induced meningioma located in the right parieto-occipital region (A) Radiation-induced parieto-occipital meningioma (red arrow) in a transverse view on MRI. (B) The same radiation-induced meningioma (red arrow) in a coronal view. As illustrated by the scale, the meningioma measures 47.9 × 40.3 × 43.0 mm.

On hospital day 16, MRI was performed for the underlying cause of seizures, and it showed extensive injury to the brain. MRI showed extensive cortical gyri form enhancement and cortical restricted diffusion. It also showed a 4.8 × 4.0 cm left temporal-parietal meningioma, likely to be radiation induced (Figures 1A, 1B).

**Figure 2:**
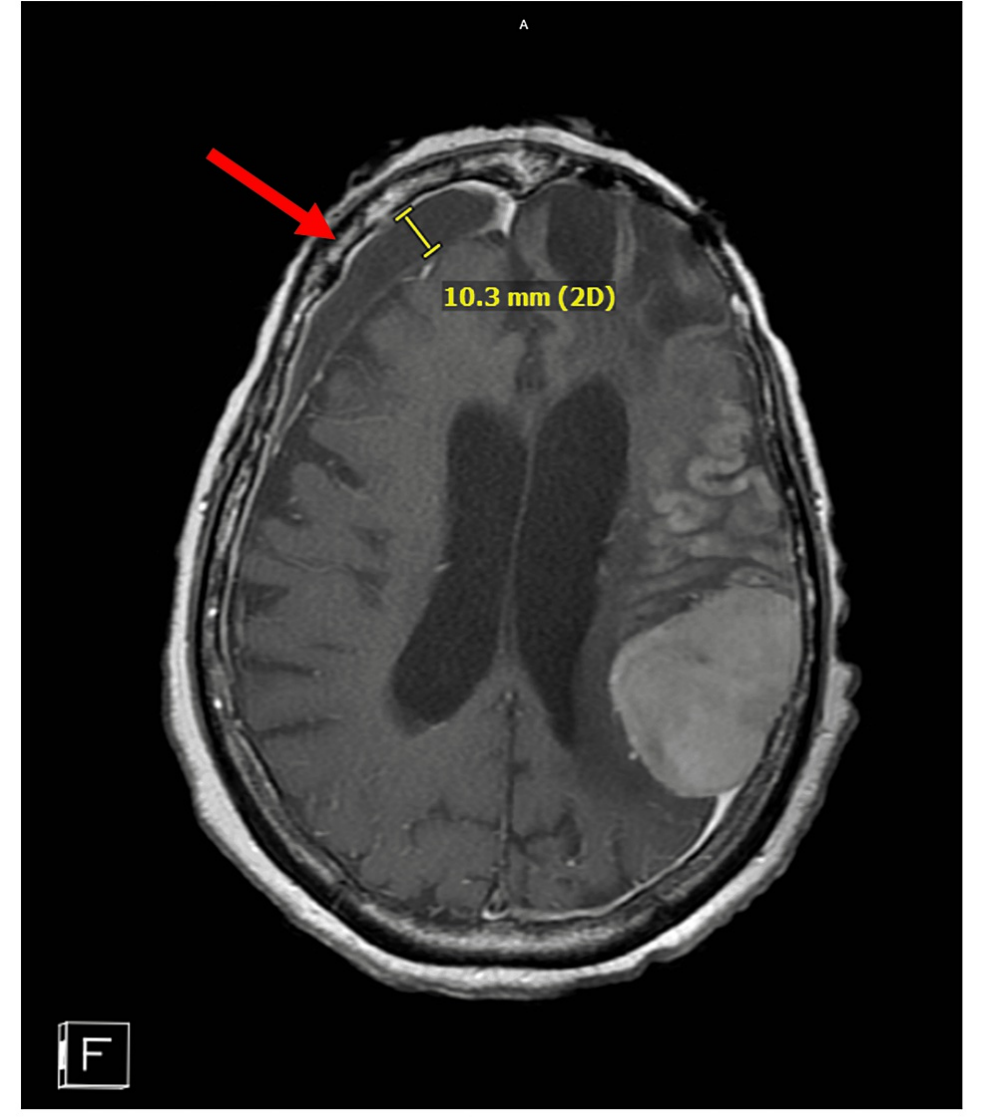
Left frontal subdural hematoma observed in the MRI scan The chronic subdural hematoma (red arrow) measures 10.3 mm as noted by the scale.

**Figure 3:**
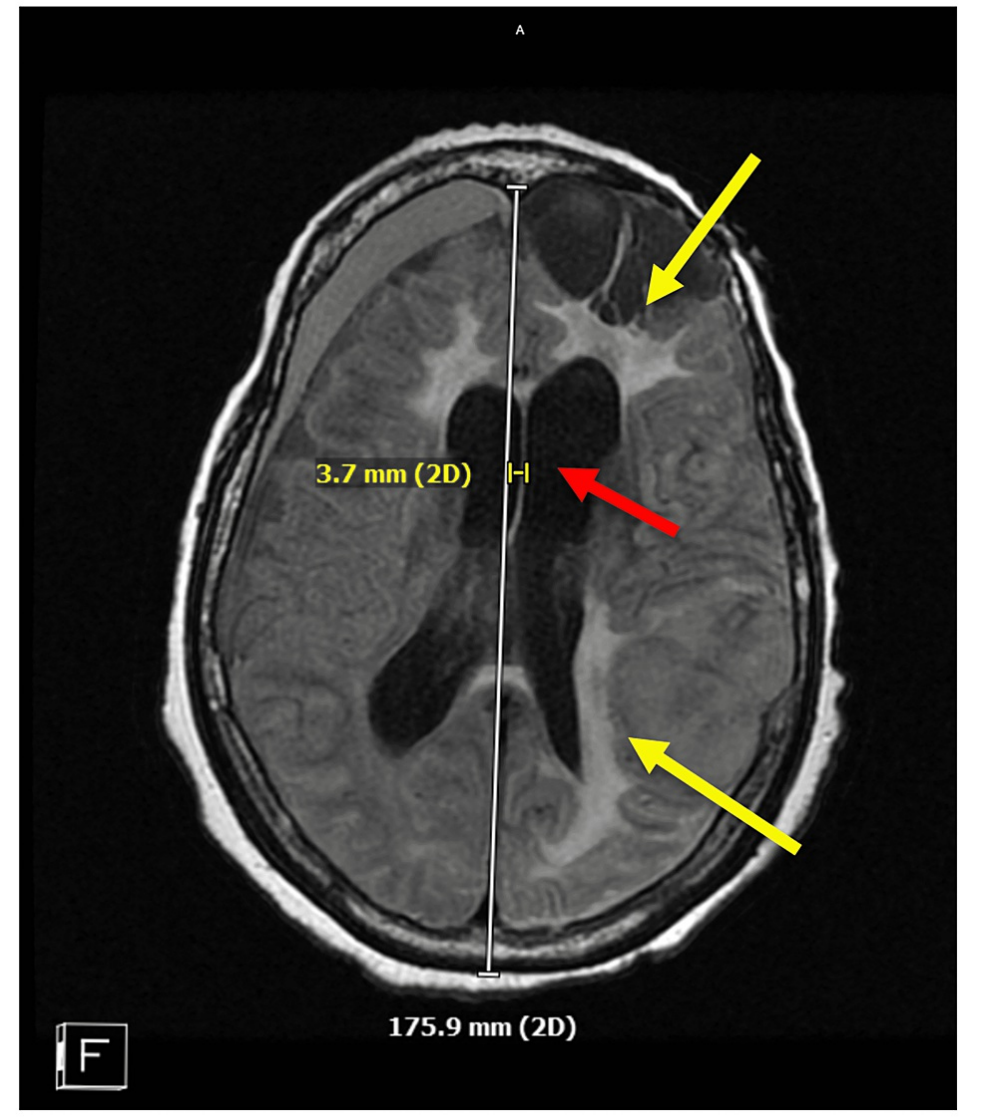
MRI of the patient's brain in transverse view demonstrating chronic changes In this transverse view, a 3.7-mm midline shift is seen (red arrow), likely from the subdural hematoma. There are also areas of edema noted (yellow arrows).

There was also extensive left inferior frontal postsurgical cystic encephalomalacia subjacent to the left frontal craniotomy flap. Small chronic right frontal subdural hematoma 1 cm in size and diffuse cerebral atrophy (advanced for age) were also noted (Figure 2). Scattered chronic microhemorrhages and midline shifts were noted (Figure 3).

The patient continued to seize through four anti-seizure medications (ASMs) and propofol. Combined with the extensive MRI findings, SMART syndrome was suspected. Magnesium was started for the potential SMART syndrome.

On hospital day 17, the patient was sedated, with no seizures. When sedation was lightened on day 18, seizures returned. Depakote 200 mg was added to the regimen. The patient continued Propofol, Keppra 1500 twice daily, Vimpat 200 mg twice daily, Onfi 20 mg twice daily, Fycompa 8 mg at bedtime, magnesium gtt. Magnesium levels were kept at 3-4 mEq/L. On hospital day 19, ketamine dose was increased to achieve a burst suppression ratio of less than 15. Since ketamine was not successful in achieving the target burst suppression, a continuous drip of Versed was added on hospital day 20. After this addition, a burst suppression ratio of less than 15 was achieved. On hospital day 20, Vimpat and Keppra were discontinued. The decision was made to wean off all sedation. Versed was weaned over the next 6-12 hours, followed by weaning of ketamine and then weaning of propofol. On hospital day 22, the patient was off all sedation, and recurrence of left occipital spikes and focal subclinical seizure was noted. At this point in time, Solu Medrol 1 g for three days and Versed gtt was added. Solu Medrol was added because of steroid response to SMART syndrome. On hospital day 23, Versed gtt was increased. On hospital day 25, the patient was off all sedation, with patient's eyes open to sternal rub, breathing with positive end-expiratory pressure, but remained hypotensive on pressors without a clear cause. On hospital day 24, no changes were noted. The patient had no clinical seizures. She remained dependent on pressors. The patient was functionally tetraplegic and poorly responsive.

## Discussion

Clinical features

The history of SMART syndrome dates back to 1995 when Shuper et al. published a case series of four children with transient “complicated migraine-like episodes” 1.2-2.8 years after cranial irradiation [9]. Bartleson et al. first postulated the existence of a novel clinical syndrome in 2003, and the SMART syndrome acronym was proposed by Black et al. in 2006 [1,3]. The diagnostic criteria proposed by Black et al. included the history of cranial irradiation, reversible neurologic dysfunction, transient gadolinium gyral enhancement and exclusion of other disorders. Commonly associated neurologic deficits include speech disturbance, visual field defects, altered mental status, hemiparesis and chemosensory deficits [1,2]. One recent report called into question the reversibility of these deficits [2]. Headaches and seizures are present in the majority of cases [5,6]. MRI findings of unilateral cortical thickening, hyperintensity and gadolinium enhancement in a gyral pattern are highly characteristic [5]. MRI findings are transient and have a predilection for the parieto-occipital cortex and sparing of the white matter [3,10].

Pathophysiology

The pathophysiology of SMART syndrome remains a subject of debate. There are two proposed, non-exclusive mechanisms [1,4]. The first involves a delayed effect of radiation on the cerebral vasculature, which is thought to impair autoregulation and disrupt the blood-brain-barrier (BBB), leading to hyperperfusion, neurologic dysfunction and enhancement on MRI. This is similar in mechanism to posterior reversible encephalopathy syndrome (PRES), and is consistent with a parieto-occipital distribution, as this area is hypothesized to be vulnerable to radiation damage [4]. The alternative explanation hypothesizes that radiation affects the trigeminovascular system, key to migraine pathogenesis. This in turn reduces the threshold to cortical spreading depression, leading to migrainous aura and possibly seizures [11].

Role of seizures

While seizures are commonly described in patients with SMART syndrome, their prevalence and role in the pathogenesis and clinical syndrome are unclear. The presence of some type of documented seizure activity is as high as 82% (9 of 11 cases) or 83% (5 of 6 cases) [2,11]. Many of these patients had electrographic seizures without clinical seizure activity or had seizures diagnosed only with long-term video EEG monitoring. Thus, in the absence of long-term monitoring, it is likely that many published reports underdiagnose the prevalence of seizures in SMART syndrome. Furthermore, the MRI signs of SMART syndrome are highly similar to post-ictal changes, and indeed, seizures without SMART syndrome are an important differential diagnosis [10].

Although seizures are common, refractory status epilepticus in SMART syndrome is rare, with only isolated cases reported. Only one of these reported cases had SRSE, which required continuous infusions of midazolam and ketamine to control, as in our patient [12]. In a cohort of five patients with status epilepticus, mostly non-convulsive, subdural fluid collections were noted in three, and a comorbid febrile respiratory infection was also noted in three [11]. Our patient initially presented with septic shock due to a complicated, febrile urinary tract infection, and was also found to have an incidental subdural hematoma. The evidence is insufficient to associate either of these clinical findings with her presentation, but it does raise the intriguing possibility that these additional insults, such as newly diagnosed meningioma, could trigger the onset of SMART syndrome decades after radiotherapy.

The high prevalence of seizures in SMART syndrome, and the similarity of MRI findings raise the question of whether seizures are an integral part of the pathogenesis and presentation of SMART syndrome. Previous reports have commented on the possible confabulation of cause and effect between the two [4,11]. Are parieto-occipital cortical damage and hyperperfusion the result of radiation, in turn predisposing the patient to seizures? Or are recurrent seizures a cause of the cortical abnormalities and neurologic deficits seen in SMART syndrome? These questions remained unanswered for now. In the rare instances where brain biopsy was performed, no pathology was demonstrated [2]. Functional studies, such as positron emission tomography or single-photon emission computerized tomography, may provide more insight into the dynamic neurovascular changes underlying the complex interplay between SMART syndrome and seizures.

## Conclusions

SMART syndrome is a rare complication of radiation therapy to the brain. As seen in our patient, SMART syndrome typically develops decades after radiation treatment. SMART can present with a variety of symptoms, including migraines, neurological deficits and encephalopathy. Recent cases of SMART syndrome demonstrate that seizures may be a hallmark feature. Our case contributes to this growing number of cases that demonstrates that SMART syndrome can present with SRSE. We hope that this case would help future providers diagnose and manage patients with a history of radiation therapy who present with neurological dysfunction.
